# A single-photon source based on topological bulk cavity

**DOI:** 10.1038/s41377-025-01929-4

**Published:** 2025-08-28

**Authors:** Xin-Rui Mao, Wei-Jie Ji, Shao-Lei Wang, Han-Qing Liu, Bang Wu, Xu-Jie Wang, Li Liu, Lai Zhou, Haiqiao Ni, Zhichuan Niu, Zhiliang Yuan

**Affiliations:** 1 Beijing Academy of Quantum Information Sciences, Beijing, 100193 China; 2 National Key Laboratory of Microwave Imaging Technology, Aerospace Information Research Institute, Chinese Academy of Sciences, Beijing, 100190 China; 3 Center of Materials Science and Optoelectronics Engineering, University of Chinese Academy of Sciences, Beijing, 100049 China; 4 State Key Laboratory of Optoelectronic Materials, Institute of Semiconductors, Chinese Academy of Sciences, Beijing, 100083 China

**Keywords:** Single photons and quantum effects, Quantum dots

## Abstract

Topological photonics offers the potential to develop quantum light sources with inherent robustness against structural disorders. To date, topologically protected edge or corner states have been investigated for this purpose. Here, for the first time, we exploit a topological bulk state with vertical directionality to enhance the light emission from a single semiconductor quantum dot (QD). An irregular ‘Q’-shaped cavity is applied for establishing topological robustness. We experimentally demonstrate a 1.6-fold Purcell enhancement of single-photon emission in the topological bulk cavity, with tolerance to the emission wavelength or the positioning of the coupled QD. Simulations indicate that such a QD-cavity coupling system can retain a Purcell factor exceeding 1.6 under a broad spectral detuning range of 8.6 nm or a coverage area of 2.5 μm^2^. Furthermore, the optimized cavity structure integrated with a reflector predicts a high single-photon extraction efficiency up to 92%. Our results offer a novel approach to develop topologically protected quantum light sources with high extraction efficiency and robust QD-cavity interaction against irregular cavity boundaries.

## Abstract (web summary)

We demonstrate a single-photon source based on topological bulk cavity, achieving high extraction efficiency and robust QD-cavity interaction against irregular cavity boundaries.

## Graphical abstract

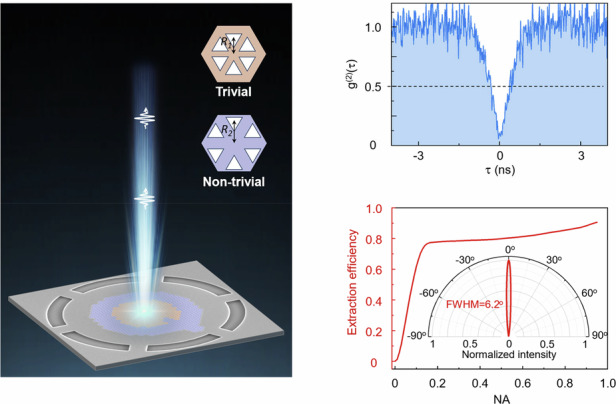

## Introduction

Single photons have emerged as a promising resource for encoding quantum bits in various quantum information applications, including quantum communication^1^, photonic quantum simulation^2^, photonic quantum sensing^3^ and optical quantum computing^4^. Single-photon sources based on self-assembled semiconductor quantum dots (QDs) serve as attractive candidates for scalable solid-state quantum information platforms^5–9^, offering superior advantages such as ultra-brightness and seamless interconnectivity with matter qubits. By coupling a single QD to an engineered photonic structure, deterministic generation of high-quality single photons have been demonstrated through cavity quantum electrodynamics (CQED) effect^10–12^. Various optical micro/nano-structures, including micropillar^13–15^, open-microcavity^16,17^, circular Bragg grating cavity^15,18,19^, 2D photonic crystal (PC) defect cavity^20,21^ and 1D PC waveguide^22–25^, have been explored as promising platform for realizing high-performance single-photon sources with simultaneously high efficiency, purity and indistinguishability. However, light-matter interaction at the micro/nanoscale is highly sensitive to defects or disorders, which may significantly degrade the device performance. Additionally, high-precision QD–cavity coupling at micro/nanoscale is often a low-probability event due to spatial and spectral mismatches between single QDs and optical modes. Complex QD positioning technologies such as wide-field photoluminescence (PL) imaging and super-resolved snapshot hyperspectral imaging have been utilized to achieve efficient light–matter interactions^26,27^.

The topological quantum optics interface, which couples quantum emitters with topologically protected cavity modes, provides a platform to construct quantum light sources with inherent topological robustness against perturbations such as defects or disorders^28–30^. To date, topologically protected quantum emitters in 0D^31^ and 1D edge states^32–38^ as well as 0D corner states^39,40^ have been experimentally demonstrated, revealing novel phenomena such as chiral quantum interfaces and Purcell enhancement in topologically nontrivial region. Topological interface effects have garnered significant attention, the bulk properties of topological structures, however, remain underexplored in the realm of quantum optics. Furthermore, extraction efficiency of topologically protected single-photon sources has received little attention and has not been adequately discussed. Recently, bulk topological effect has been employed in laser physics^41–43^. A bulk state can be laterally confined within the topological interface due to band-inversion-induced reflection at the Γ point of Brillouin zone, which gives rise to high-performance single-mode lasing with directional vertical emission^41^. This new type of topological bulk laser indicates the potential to exploit topological bulk properties in related fields, including quantum photonics.

Here, for the first time, we design and demonstrate a single-photon source by coupling a single InAs/GaAs QD to a topological bulk cavity. The topological bulk cavity is formed by two types of PC distinct in topology. Bulk states in the cavity are confined by band-inversion-induced reflection and exhibit out-of-plane directionality around the Γ point. This configuration facilitates the realization of single-photon sources with high extraction efficiency. The low quality-factor (Q) and extended mode area of the bulk state result in tolerance to detuning of the QD emission wavelength and large area for effective light-matter interaction. The simulated Purcell factor (*F*_p_) exceeds 1.6 under a broad spectral range of 8.6 nm or a coverage area of 2.5 μm^2^. Purcell effect has been observed in time-resolved lifetime measurements, demonstrating an enhancement of the QD emission rate by a factor of 1.6. Second-order correlation measurements further confirm the single-photon nature of the QD emission through the observation of anti-bunching. Furthermore, our calculation shows that the single-photon extraction efficiency of the topological bulk cavity on a reflector can reach up to 77% at a modest numerical aperture (NA) of 0.15. The comparison between our work and other quantum light sources based on topological PC is shown in Supplementary Information Table S1. Our results indicate the potential applications of topological bulk effect in developing robust quantum sources with high extraction efficiency.

## Results

### Design of single-photon source based on topological bulk state

**Fig. 1:**
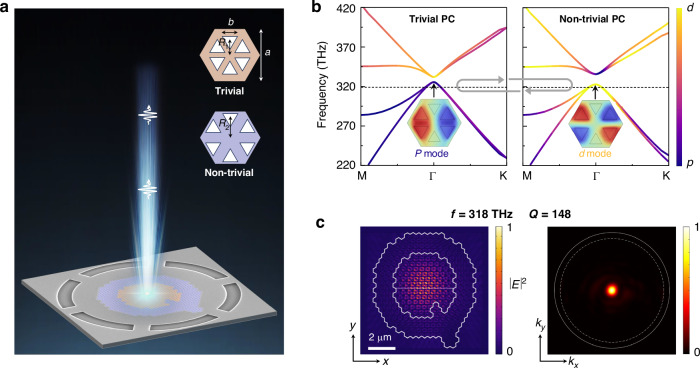
**Design of the single-photon source based on a topological bulk cavity.** **a** Schematic of the single-photon source, featuring a single quantum dot (QD) coupled to a ‘Q’-shaped topological bulk cavity. Inset: Unit cells of trivial and non-trivial PCs. **b** Band structures of the topological trivial (left panel) and non-trivial (right panel) PCs, where *a* = 420 nm, *b* = 123 nm, *R*_1_ = 0.97×*a*/3, and *R*_2_ = 1.048×*a*/3. The hybridization between *p* and *d* bands is indicated by the color scale. Inset: *p* and *d* modes with opposite parities at Γ points of the lower bands in these two PCs. **c** Simulated near-field (left panel) and angle-resolved far-field (right panel) distribution of dipole-like bulk state in the ‘Q’-shaped cavity. White lines in the left panel indicate the topological interface. Solid and dashed circles in the right panel indicate the light cone and NA ( = 0.9) of the collection objective, respectively. The eigen-frequency of the bulk state is displayed by dashed lines in Fig. 1b

Figure 1 provides a detailed illustration of the design and operation principle of the single-photon source utilizing a topological bulk cavity. As shown in Fig. 1a, a single QD is coupled to a topological bulk state and the single-photon emission from the QD-cavity system exhibits out-of-plane vertical directionality. The bulk cavity is constructed by a topologically non-trivial PC encircling a topologically trivial PC. The non-trivial or trivial PCs are achieved by moving the triangle nanoholes away from or towards centers of unite cells in the same honeycomb lattice, respectively. Three-dimensional full wave simulations are carried out to obtain band structures and optical modes of the topological cavity (see Material and methods). The simulated bulk band dispersions are presented in Fig. 1b, where the dipole (*p*)/quadrupole (*d*) mode component of the bands is indicated by the color scale. At frequency around the Γ point, the bulk modes in the trivial and non-trivial PC possess opposite parities, leading to the band-inversion induced reflection (grey arrows in Fig. 1b). As a result, light waves around the Γ point get reflected at the topological interface and can be laterally confined in the topological cavity^41^.

The topological bulk states confined by band-inversion-induced reflection are robust against cavity deformations when adding or removing unit cells of the non-trivial or trivial PCs. Accordingly, we design an ‘Q’-shaped cavity with an irregular cavity contour to verify the robustness of the bulk states (Fig. 1a–c). The near-field and far-field distributions of a dipole-like bulk state in the ‘Q’-shaped topological cavity is shown in Fig. 1c. In the near field, the bulk state is well confined within the irregular topological boundaries with field intensities spreading over the trivial PC. In the far field, the in-plane momentum of the bulk state is pinned around the Γ point. Apart from this dipole-like bulk state, one edge state and one quadrupole-like bulk state also exist in the ‘Q’-shaped topological cavity (Supplementary Information Figure S1). Notably, the dipole-like bulk state exhibits a low quality-factor of 148 and far-field directionality with a divergence angle less than 8^o^ (Supplementary Information Figure S2), which is advantageous for developing flexible and robust single-photon sources with broadband Purcell enhancement and vertical emission directionality.

### Fabrication and characterization of an irregular bulk cavity

**Fig. 2:**
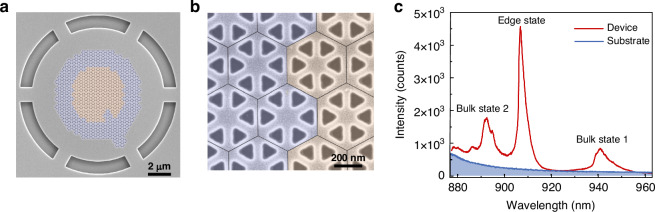
**Fabricated device and characterization of the topological cavity modes.** **a** SEM image of a fabricated ‘Q’-shaped topological bulk cavity patterned on a GaAs wafer. The cavity is composed of a trivial PC (orange) encircled by a topological PC (purple). **b** Enlarged SEM image of the device. Black hexagons indicate unit cells of the PCs. **c** PL spectra collected from the device or substrate under high pump power. Three peaks correspond to the three topological states in the cavity, as shown in Fig. 1c and Figure S3. Bulk state 1 and bulk state 2 correspond to the dipole-like bulk state and the quadrupole bulk state, respectively

We fabricate the designed structures on a 170-nm-thick GaAs slab with a monolayer of self-assembled InAs QDs grown by molecular beam epitaxy. Suspended devices are obtained using electron beam lithography, inductively coupled plasma etching and finally wet etching, as detailed in Supplementary Information Fig. S3. We note that further surface passivation could be a viable approach to reduce the adverse influence induced by the etched surfaces and optimize QD optical performance^37,44^. Figure 2a and b shows the scanning electron microscopy (SEM) images of a fabricated device. The common lattice constant *a* for both the trivial and non-trivial PCs is 420 nm. For these two PCs, distances from the center of hexagonal unit cell to the center of each triangular nanohole (side length *b* ~ 123 nm) are *R*_1_ = 0.97×*a*/3 (trivial) and *R*_2_ = 1.048×*a*/3 (non-trivial), respectively.

Confocal PL measurement is performed to characterize the cavity-mode. The sample is placed inside a cryostat maintained at temperature of 4 K, and a 780-nm continuous-wave (CW) pump laser is used to excite the device (Supplementary Information Fig. S4). Under a high pump power of 43.6 μW, the PL spectrum reveals three distinct peaks as shown in Fig. 2c, corresponding to three eigenstates in the ‘Q’-shaped topological cavity. The dipole-like bulk state near the band edge of the lower bulk bands (Figs. 1b and S3a) corresponds to the peak at wavelengths of 940.85 nm. While PL spectrum of the substrate exhibits no discernible peaks within the focused wavelength region. The measured quality-factor of the dipole-like bulk state is approximately 120, which matches well with the simulated result presented in Fig. 1c.

### Single-photon emission from the bulk cavity

**Fig. 3:**
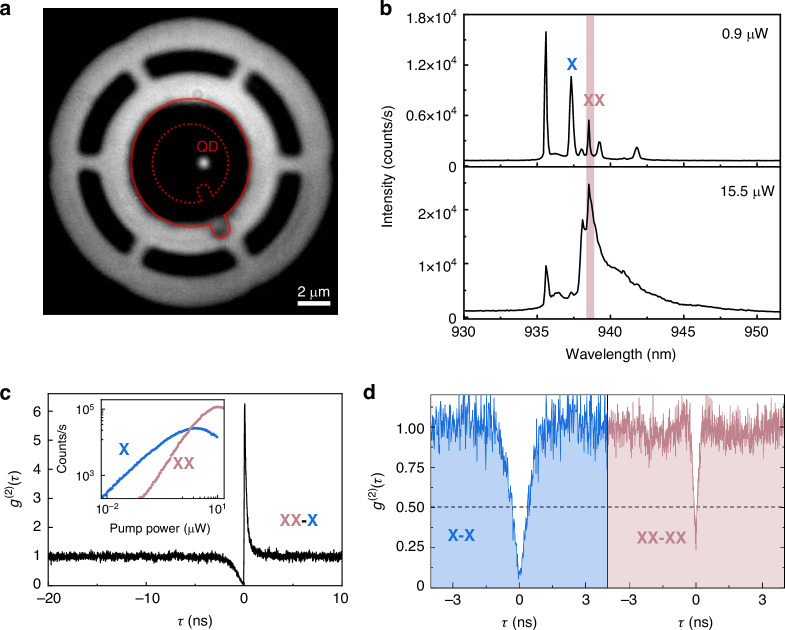
**Single-photon emission from the topological bulk cavity.** **a** Image of the device with a single QD located inside a ‘Q’-shaped topological bulk cavity. The device is illuminated by a halogen lamp and simultaneously the QD is excited by a 780 nm CW laser. The red dashed line indicates the topological interface, and the red solid line indicates the outer contour of the cavity. **b** PL spectra measured under different pump power. **c** Cross-correlation measurement of photons emitted from the XX–X cascade. Inset: Logarithmic plot of X and XX count rates as a function of excitation power. **d** Auto-correlation measurement for X or XX photons

To illustrate the behavior of single-photon emitting based on topological bulk cavity, we pump the device at low excitation powers and investigate the radiation properties of a single QD in the cavity. The fluorescence image in Fig. 3a confirms that the QD is situated inside the bulk cavity, with a deviation of 0.8 μm from the cavity center. PL spectra of the device at different pump powers are shown in Fig. 3b. At a low pump power of 0.9 µW, several transition lines of the QD can be identified in the spectrum, including exciton (X) and biexciton (XX) emissions (Supplementary Information Fig. S6). When the pump power is increased to 15.5 µW and sufficient to excite bulk state, we observe that XX emission of the QD is resonant with this cavity mode, exhibiting maximum intensity compared to other transitions peaks.

Figure 3c shows the result of XX-X cross-correlation measurement, which initiates with XX photons and terminates with X photons. The asymmetric bunching effect observed at positive delay times confirms the XX-X cascaded emission of photon pairs within the same decay channel. Additionally, the dependence of measured photon count rates on excitation power is depicted in Fig. 3c. As the pump power increases, the PL intensity of the cavity-enhanced XX emission gradually surpasses that of the X emission. The slopes of X and XX emissions in the log-log scale are 0.98 and 1.5, respectively. We note that the XX exponent being less than 2 may be attributed to the presence of non-radiative recombination processes^45^. As shown in Fig. 3d, the single photon nature of X or XX emissions from the topological bulk cavity is demonstrated through second-order auto-correlation measurements, revealing low multi-photon probabilities of g^(2)^(0)_X_ = 0.05 and g^(2)^(0)_XX_ = 0.24 (raw data).

### Purcell effect of single QD in the bulk cavity

As discussed in Fig. 1c, the dipole-like bulk state in the topological cavity has a low *Q* factor of ~10^2^, which facilitates broadband Purcell enhancement. Additionally, the bulk state is confined by band-inversion-induced reflection and the in-plane mode extension is restricted by the topological interface. Consequently, the area of real-space field distribution is only determined by periods of the intracavity PC, facilitating deterministic QD-cavity coupling without complex QD-positioning techniques (Supplementary Information Fig. S7). As a result, QD-cavity coupling in the topological bulk cavity is tolerant to both emission wavelength and relative position of the QD.

**Fig. 4:**
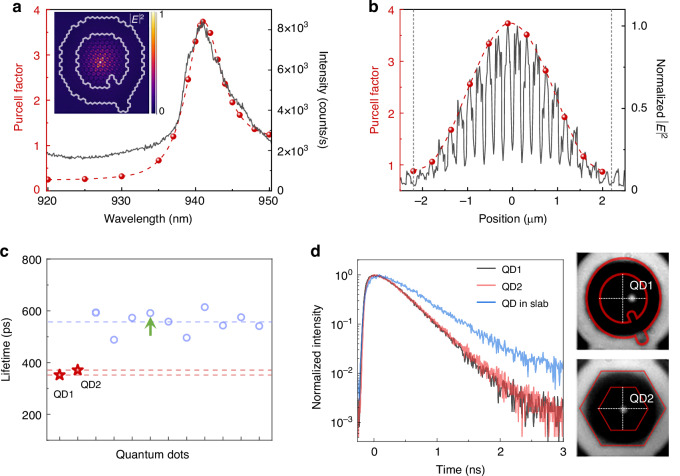
**Frequency- and position-dependent Purcell effect in topological bulk cavity.** **a** Simulated Purcell factor of bulk state versus emission wavelength (red dots). The PL spectrum of bulk state from Fig. 2c is also shown for comparison (black curve). Inset: Simulated radiation field (|**E** | ^2^) from a single emitter coupled to bulk state. **b** Simulated Purcell factor of bulk state versus source position (red dots) and the corresponding electric field intensity distribution (black curve) along the white dashed line in Fig. 1c. **c** The measured radiative lifetimes of XX photons from QD1 and QD2 coupled to topological bulk cavities (red) compared to the lifetimes of XX photons from QDs in slab (blue). **d** Left panel: Time-resolved XX photon counts of QD1, QD2 and a QD in slab (indicated by green arrow in Fig. 4c). Right panel: Position of QD1 and QD2 in relative to the topological bulk cavity

The simulated Purcell factor for this device at different emission wavelengths of a single emitter is shown in Fig. 4a (see Material and methods). The result reveals spontaneous emission enhancement (*F*_p_ > 1) around the bulk state at 941 nm, as well as spontaneous emission inhibition (*F*_p_ < 1) within the photonic band gap at shorter wavelengths. When the emitter is resonant with the bulk state, the maximum *F*_p_ is calculated to be 3.7. Owing to the low Q-factor, such a wavelength-dependent *F*_p_ has a full-width at half-maximum of approximately 7 nm, which matches well with the measured PL spectrum of the cavity mode. Figure 4b presents simulated *F*_p_ of the bulk state with varied single-emitter positions, where different position corresponds to different field intensity of the cavity mode. The simulation results reveal that the QD-cavity coupled system can sustain Purcell factors >1.6 over an area of 2.5 μm². As discussed in Supplementary Information Figs. S7 and S8, the topological bulk mode exhibits a position-dependent local distribution with an extended field envelope, enabling a statistically significant probability of observing pronounced Purcell enhancement. As a result, the insensitivity of QD positioning in the bulk cavity can be further improved by simply increasing the number of periods of intracavity lattice (trivial PC used in this work). We note that while the mode volume (*V*_m_) increases with larger PC period number, the *Q*-factor also rises due to the enhanced in-plane confinement. Consequently, the ratio of *Q*/*V*_m_, which governs the Purcell enhancement, increases with larger PC period (Supplementary Information Fig. S7).

Using a 780 nm pulsed laser (5 ps pulse width, 80 MHz repetition rate) for above-band excitation, we conducted time-resolved fluorescence measurements, as shown in Fig. 4c and d. The results indicate that the XX emission radiative lifetimes of two single QDs coupled to two distinct topological bulk cavities are 352 ps (QD1) and 371 ps (QD2). These lifetimes are approximately 1.6 and 1.5 times shorter, respectively, than the average XX lifetime of ~10 QDs from the same area in the slab. Simulated near-field and far-field properties of bulk state in these two cavities are shown in Fig. S4. Although QD1 and QD2 are coupled to topological bulk cavities with different geometries respectively and their relative positions to the cavity modes also differ from each other (Fig. 4d), the radiation properties of these two QD are similarly modulated by the topological cavities.

### Extraction efficiency of an optimized bulk cavity

**Fig. 5:**
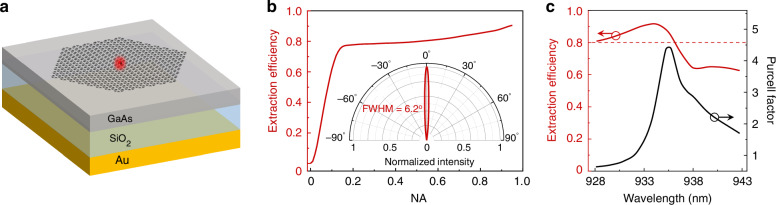
**Extraction efficiency of the optimized single-photon source with a reflector.** **a** Schematic of the optimized single photon source with a GaAs/SiO_2_/Au structure. **b** Simulated extraction efficiency of the optimized single-photon source as a function of NA. Inset: Angle-resolved far-field |**E** | 2 distribution of the optimized structure. **c** Simulated extraction efficiency and Purcell factor of the optimized structure versus emission wavelength. In the simulations, the extraction efficiency is defined as the ratio of the radiated power collected within the NA to the total radiated power in the full solid angle, from a single emitter embedded in the topological cavity

Band-inversion-induced reflection in the topological cavity only occurs around the Γ point. Thus, the bulk state exhibits out-of-plane directionality with small divergence angle, which can significantly improve the single-photon extraction efficiency. The simulated extraction efficiency of an optimized topological bulk cavity integrated with a reflector is shown in Fig. 5. *R*_1_ and *R*_2_ of the optimized topological structure are set to be 0.92×*a*/3 and 1.05×*a*/3 respectively, where the lattice constant *a* is 420 nm (Supplementary Information Fig. S9). As shown in Fig. 5a, the GaAs cavity is sitting on a highly efficient reflector consisting of 300-nm SiO_2_ and 200-nm gold (Au) to effectively suppress the downwards photon leakage. For a single QD coupled to the topological bulk cavity, the simulated out-of-plane directionality and extraction efficiency at the first lens are plotted in Fig. 5b. The results indicate that a nearly Gaussian far-field distribution (Supplementary Information Fig. S9) and the divergence angle of the radiation field is only 6.2°. The simulated extraction efficiency is as high as 92% and can reach 77% at a small NA of 0.15. Figure 5c shows the tolerance of the extraction efficiency to excitation wavelength, where the extraction efficiency retains above 80% over a wavelength range of 8 nm.

## Discussion

In conclusion, we have demonstrated a novel single-photon source based on a single QD in topological bulk cavity. The dipole-like bulk state in the topological cavity features a low *Q* factor and an adjustable mode size determined solely by the topological interface, resulting in a broadband Purcell effect and insensitivity of QD positioning during light-matter interaction. Time-resolved PL spectroscopy demonstrates a Purcell factor of 1.6 for a single QD resonant with an irregular ‘Q’-shaped topological cavity. The anti-bunching behavior of single photon emission is verified by second-order auto-correlation measurements. Moreover, as the band-inversion-induced confinement occurs around the Γ point, this single-photon source exhibits directional vertical emission with a simulated extraction efficiency of 92%. Our results introduce a novel approach for the development of topologically protected quantum light sources with high extraction efficiency and robust QD-cavity interaction against irregular cavity boundaries. In addition, such a single-photon source is compatible with electrical connections, which can enable a new type of charge-tunable quantum light sources without the need for additional integrated straight bridges, as required in electrically-driven circular Bragg grating^46^.

## Materials and methods

### Full-wave simulation

By using the commercial software of COMSOL Multiphysics based on finite-element method, three-dimensional full wave simulations are carried out to obtain band structures of the trivial and non-trivial PCs, optical modes of the topological cavity, and also the emission properties of a single emitter in the cavity. The refractive index of GaAs material is set to be 3.50, corresponding to typical values near the QD emission wavelength. Perfectly matched layer domains are used to reduce the reflection from the simulation boundaries. For emitter-cavity coupling simulations, a single dipole emitter is positioned at the point of maximum cavity-field intensity, with its dipole orientation aligned parallel to the local electric field vector.

### Device fabrication

The QD wafer grown by molecular beam epitaxy comprises a 170-nm-thick GaAs membrane with InAs QDs in the center, a 300-nm-thick Al_0.8_Ga_0.2_As sacrificial layer and a 350-μm-thick GaAs substrate. Firstly, 380-nm electron-beam resist (ZEP520A) is spin coated on the QD wafer and baked at 180^o^C for 3 minutes. Secondly, we perform e-beam lithography to transfer the designed PC patterns onto the resist. Subsequently, the structures are constructed through an inductively coupled plasma etching system with Cl_2_/BCl_3_/Ar gas to etch holes in the QD layer. The residual e-beam resist is removed by oxygen plasma cleaning. Finally, 6% HF is used to etch away the Al_0.8_Ga_0.2_As sacrificial layer to form a suspended membrane (Supplementary Information Figure S3).

## Supplementary information

Supplementary Information

## Data Availability

The data that support the findings of this study are available from the corresponding author upon reasonable request.
