# Supplementary material for: A single-photon source based on topological bulk cavity
Source: Light Sci Appl. 2025 Aug 28;14:295. doi: 10.1038/s41377-025-01929-4 (PMC12394714; doi:10.1038/s41377-025-01929-4)
Supplement: Supplementary file 1 — Supplementary Information [file 41377_2025_1929_MOESM1_ESM.pdf]

Supplementary Information for

A single-photon source based on topological bulk cavity

Xin-Rui Mao[1ǂ], Wei-Jie Ji[1ǂ], Shao-Lei Wang[2ǂ], Han-Qing Liu[3,4], Bang Wu[1*], Xu-Jie Wang[1], Li Liu[1], Lai Zhou[1], Haiqiao Ni[3,4], Zhichuan Niu[3,4], Zhiliang Yuan[1]

[1] Beijing Academy of Quantum Information Sciences, Beijing 100193, China
[2] National Key Laboratory of Microwave Imaging Technology, Aerospace Information Research Institute, Chinese Academy of Sciences, Beijing 100190, China
[3] Center of Materials Science and Optoelectronics Engineering, University of Chinese Academy of Sciences, Beijing 100049, China.
[4] Key Laboratory of Optoelectronic Materials and Devices, Institute of Semiconductors, Chinese Academy of Sciences, Beijing 100083, China
ǂ These authors contributed equally to this work.
* Correspondence should be addressed to wubang@baqis.ac.cn.

Content

S1. Comparison with other quantum light sources based on topological photonic crystals 2

S2. Topological eigenstates in the 'Q'-shaped cavity 3

S3. Far-field directionality of topological bulk state 4

S4 Device Fabrication 5

S5. Optical characterization setup 6

S6. Quasi-resonant p-shell excitation measurements on QD2 7

S7. Polarization-resolved measurements on QD1 8

S8. Topological bulk states under varied cavity sizes 9

S9. Spatial distribution of the Purcell factor 10

S10. Topological bulk cavity integrated with highly efficient reflector 12

S1. Comparison with other quantum light sources based on topological photonic crystals

A comprehensive comparison between our work and the existing topological quantum light sources are summarized in Table R1. Prior works predominantly focused on 0D/1D topological edge states and 0D topological corner states, our work exploits the bulk properties of topological structures and achieve a new type of topologically protected single-photon source with broadband Purcell enhancement effect, vertical directionality and high extraction efficiency.

Table S1: Comparison with other quantum light sources based on topological photonic crystals

	Topological state	Vertical structure	Emission directionality	Simulated V_m	Measured Q-factor	Simulated F_p	Measured F_p	Measured $g^{(2)}(0)$
**Our work**	2D bulk	slab	Vertical (η ~92%)	20 $(\lambda/n)^3$	120	3.7	1.6	0.01-0.24
Nano Lett. **23**, 820-826 (2023)	0D edge	DBR	Vertical	65 $(\lambda/n)^3$	5500	4.7	1.8	0.365
Science **359**, 666-668 (2018)	1D edge	slab	In-plane (*Chiral*)	——	——	——	——	0.15
Phys. Rev. B **101**, 205303 (2020)	1D edge	slab	In-plane (*Chiral*)	——	4000	——	——	——
Optica **7**, 1690-1696 (2020)	1D edge	slab	In-plane (*Chiral*)	——	4000	——	——	0.09
Appl. Phys. Express **12**, 062005 (2019)	1D edge	slab	In-plane	——	——	——	——	——
Laser & Photonics Rev. **16**, 2200077 (2022)	1D edge	slab	In-plane	——	——	6	3	0.26
Phys. Rev. Appl. **16**, 014036 (2021)	1D edge	slab	——	——	8000	170	——	——
Laser & Photonics Rev. **14**, 1900425 (2020)	0D corner	slab	——	0.23 $(\lambda/n)^3$	1900	——	1.3	——
Light Sci. Appl. **13**, 19 (2024)	0D corner	slab	——	0.309 $(\lambda/n)^3$	1681	434	3.7	0.024

V_m: mode volume; F_p: Purcell factor; 0/1/2D: zero/one/two-dimensional; η: simulated extraction efficiency

S2. Topological eigenstates in the ‘Q’-shaped cavity

Within the ‘Q’-shaped topological cavity, there exists one dipole-like bulk state 1, one edge state, and one quadrupole-like bulk state 2. The near-field and far-field distributions of the edge state and bulk state 2 are shown in Fig. S1. In the near field, the bulk state 2 is well confined inside the irregular topological boundaries with field intensities spreading over the trivial photonic crystal (PC), similar to bulk state 1 in Fig. 1c. While the edge state is situated near the topological boundaries. In the far field, the in-plane momenta of these two states are also all pinned around the Γ point, similar to bulk state 1.

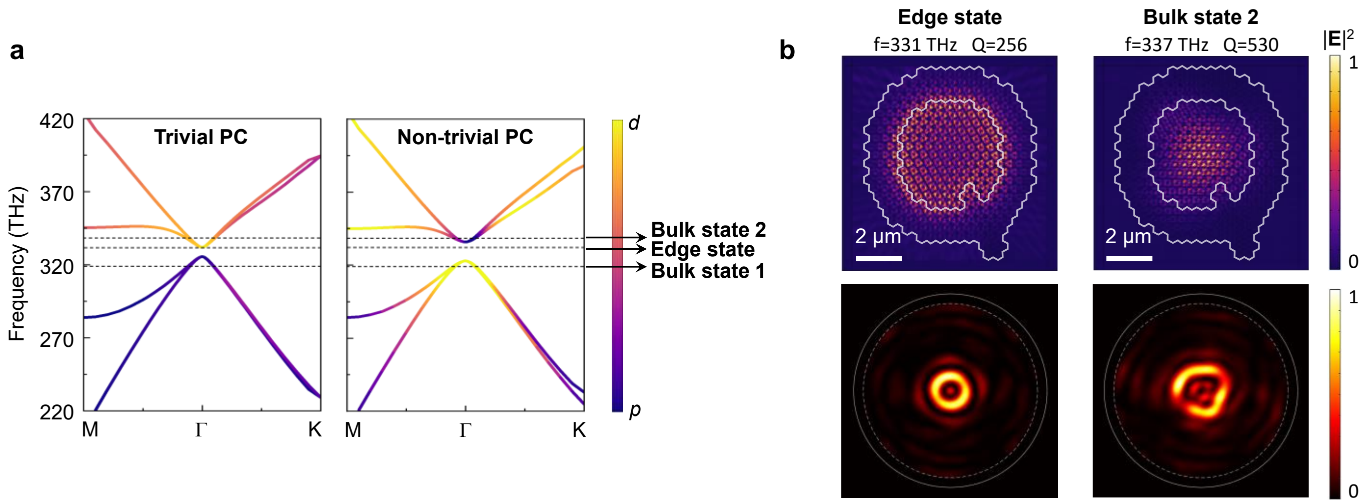

Figure S1. a Band structures in Fig. 1b. The eigen-frequencies of three topological states in the ‘Q’-shaped cavity are displayed by dashed lines. **b** Simulated near-field (top panels) and angle-resolved far-field (bottom panels) $|\mathbf{E}|^2$ distribution of the other two topological states in the ‘Q’-shaped cavity: edge state (f=331 THz, Q=256), bulk state 2 (f=337 THz, Q=530). White lines in top panels indicate the topological interface. Solid and dashed circles in bottom panels indicate the light cone and NA (=0.9) of the collection objective, respectively.

S3. Far-field directionality of topological bulk state

The near-field and far-field properties of dipole-like bulk state 1 in three topological cavities, simulated using three-dimensional (3D) full-wave, are shown in Fig. S2. The ‘Q’-shaped, hexagonal and a more irregular star-shaped topological cavities differ only in the geometry of their cavity contours. In all of these cavities, bulk state 1 is confined within the topological interface and exhibits out-of-plane directionality with a small divergence angle.

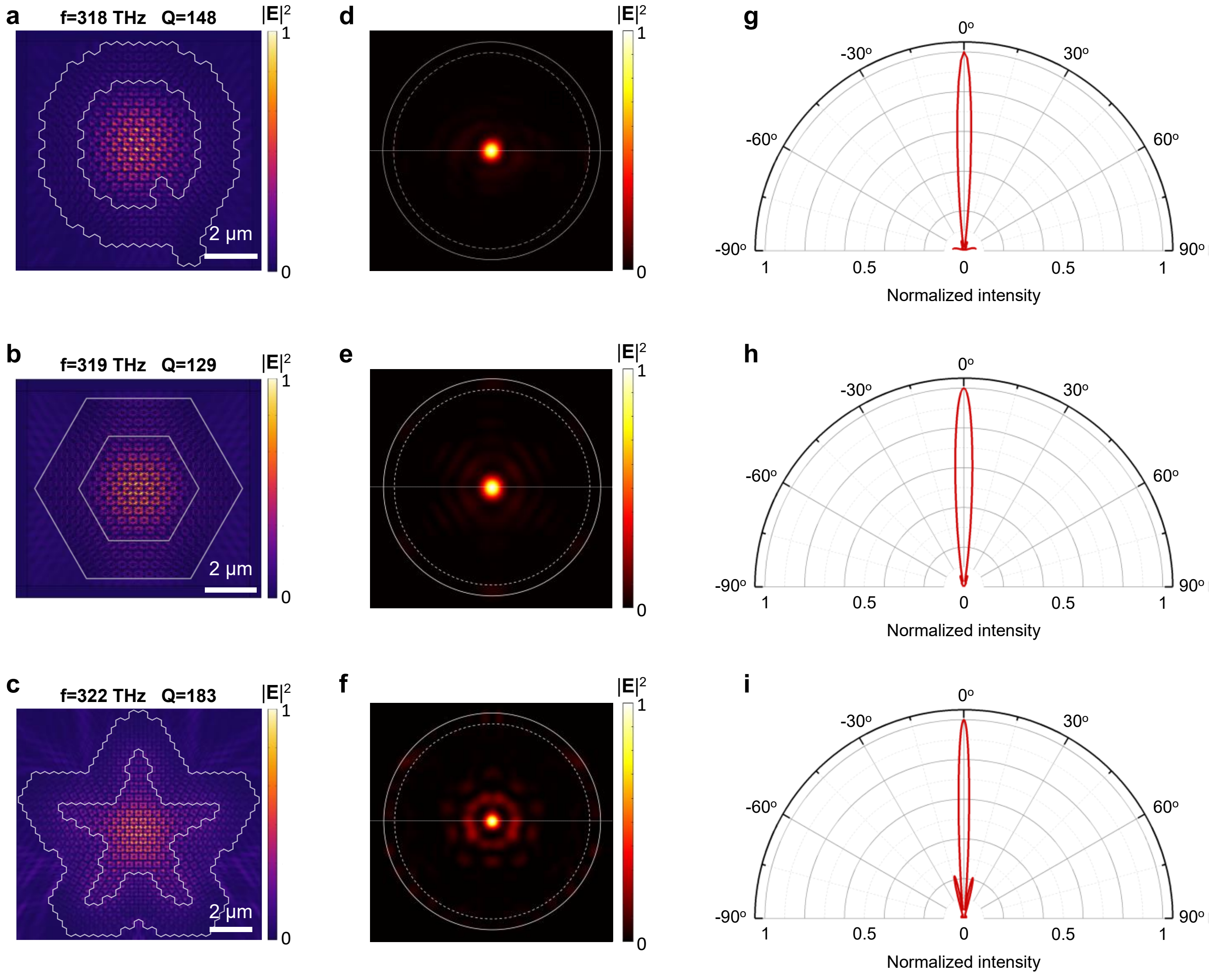

Figure S2 Vertical far-field directionality of dipole-like bulk states in topological bulk cavities with distinct cavity shape. a-c Simulated near-field $|\mathbf{E}|^2$ in the ‘Q’-shaped (**a**), hexagonal (**b**) and star-shaped (**c**) bulk cavities. **d-f** Simulated angle-resolved far-field $|\mathbf{E}|^2$ in the ‘Q’-shaped (**d**), hexagonal (**e**) and star-shaped (**f**) bulk cavities. **g-i** Simulated intensity profile along the white line in (**d-f**) in the ‘Q’-shaped (**g**), hexagonal (**h**) and star-shaped (**i**) bulk cavities.

S4 Device Fabrication

The fabrication flow is illustrated in Fig. S3. We fabricate the device on a quantum dot (QD) wafer grown by molecular beam epitaxy, which comprises a 170-nm-thick GaAs membrane with InAs QDs in the center, a 300-nm-thick $Al_{0.8}Ga_{0.2}As$ sacrificial layer and a 350-µm-thick GaAs substrate. First, we spin coat a 380-nm ZEP520A electron-beam (e-beam) resist on the QD wafer and baked at 180ºC for 3 minutes. Next, we perform e-beam lithography to transfer the designed PC patterns onto the e-beam resist. Subsequently, the structures are constructed through an inductively coupled plasma etching system with $Cl_2/BCl_3/Ar$ gas to etch holes in the QD layer. The residual e-beam resist is then removed by a gentle oxygen plasma cleaning process. Finally, 6% HF is used to etch away the $Al_{0.8}Ga_{0.2}As$ sacrificial layer to form a suspended membrane.

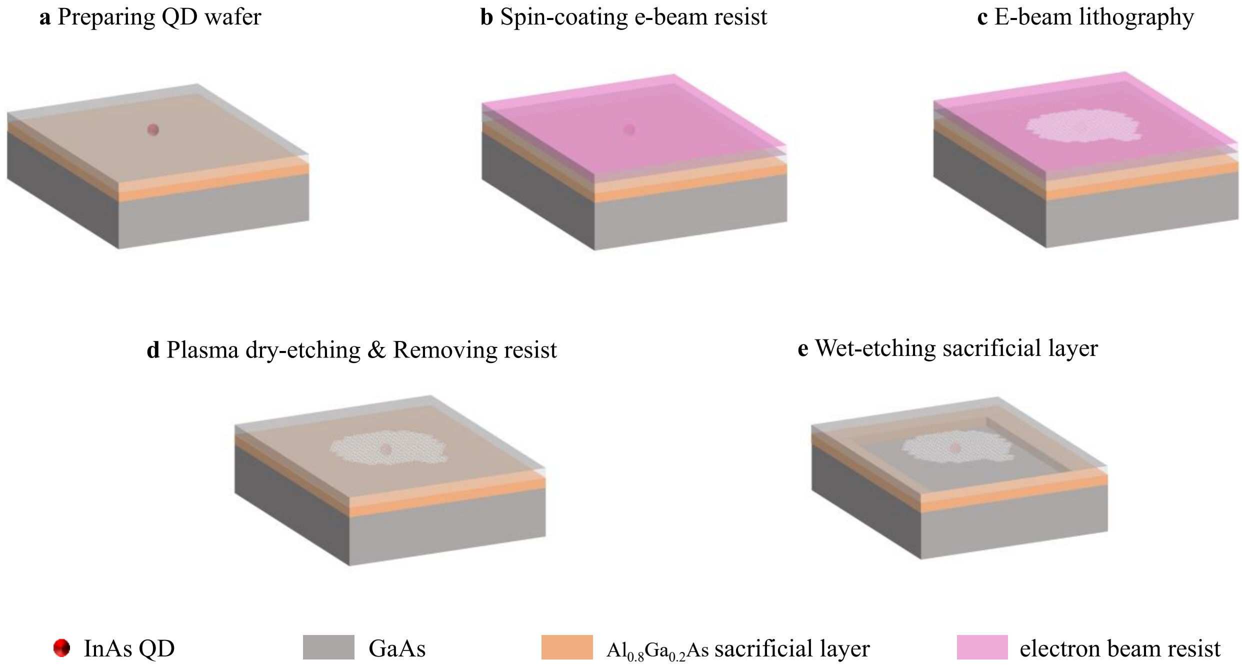

Figure S3 Flow chart of device fabrication. a Preparing the QD wafer. **b** Spin-coating e-beam resist which serves as mask. **c** Fabricating the mask by e-beam lithography to create the PC structure. **d** Transferring patterns to QD layer by dry-etching with inductively coupled plasma and then removing residual photoresist. **e** Wet-etching $Al_{0.8}Ga_{0.2}As$ sacrificial layer to form a suspended structure.

S5. Optical characterization setup

Fig. S4a illustrates the optical characterization setup. The fabricated sample is placed on a three-dimensional nanopositioner in a closed-circle cryostat, maintaining a base temperature close to 4 K. A microscope objective with a numerical aperture of 0.9 is used to focus the pump laser onto the device and to collect the emitted photons. We use a continuous-wave (CW) laser at a wavelength of 780 nm and a 80 MHz mode-locked Ti:Sapphire laser of 5 ps duration for photoluminescence (PL) and time-resolved PL measurements, respectively. A spectrometer with resolution of 0.02 nm, equipped with a liquid nitrogen cooled silicon CCD, is used to record PL spectra. The relative position between QDs and cavities are captured using an EMCCD (Electron-Multiplying CCD) camera. The spectrometer is also applied to select photons from the exciton (X) and biexciton (XX) emission lines for auto- or cross-correlation measurements. For photon counting, time-resolved or second-order correlation measurements, we employ one or two superconducting nanowire single photon detectors (SNSPDs) with 78% efficiency and 48 ps jitter, along with a time-tagger. Losses of optical components in the signal collection path are summarized in Fig. S4b.

a

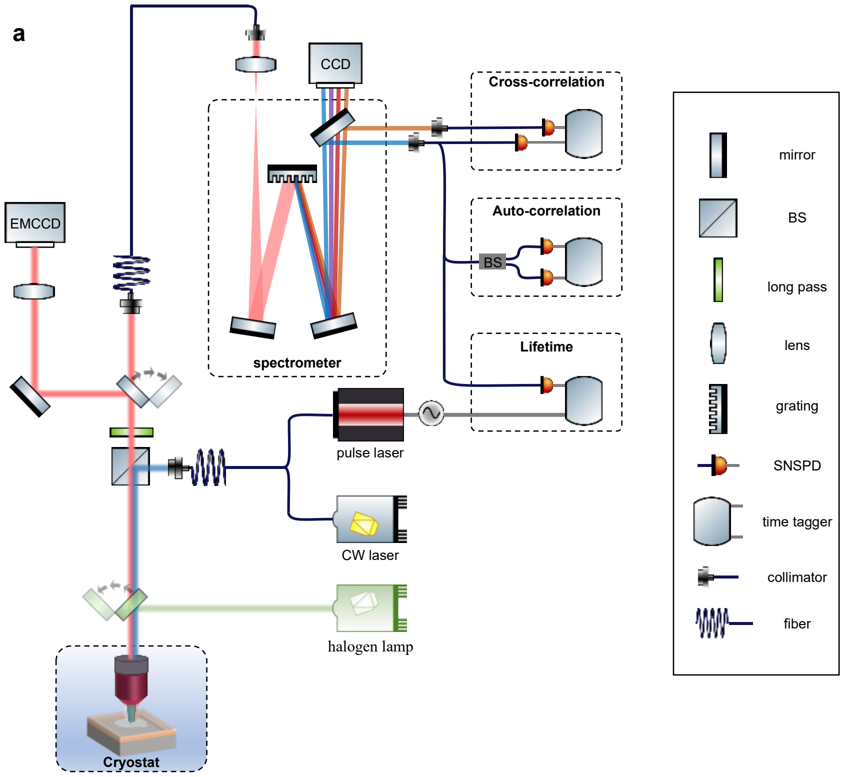

b

Optical Component	Transmission
Objective	0.67
Optical window	0.99
BS	0.46
2 Mirrors	0.95
Long pass	0.86
Fiber coupling	0.48
2 Fiber connectors	0.86
Grating filter	0.26
SNSPD	0.68
**Total transmission**	**0.018**

Figure S4. **a** Experimental setup for optical characterization: SNSPD: superconducting nanowire single photon detector. BS: beam splitter. **b** Transmission efficiency of each optical component in the collecting light path.

S6. Quasi-resonant p-shell excitation measurements on QD2

We implement a quasi-resonant *p*-shell excitation strategy. Using the QD2 device as a representative example, Figure S5a shows the *s*-shell transition at 907 nm in the PL spectrum. The photoluminescence excitation (PLE) spectrum (Fig. S5b), obtained by monitoring the *s*-shell emission intensity versus excitation wavelength, reveals a distinct resonance at 887.4 nm, which we assign to the *p*-shell transition. Under CW *p*-shell excitation, power-dependent count-rate measurements (Fig. S5c) exhibit a two-fold increase at saturation compared to above-bandgap excitation at 780 nm (Fig. S5e) owing to suppressed non-radiative recombination. Notably, the second-order autocorrelation measurements show similar single-photon purity ($g^{(2)}(0) < 0.02$) under both excitation regimes (Fig. S5d,f).

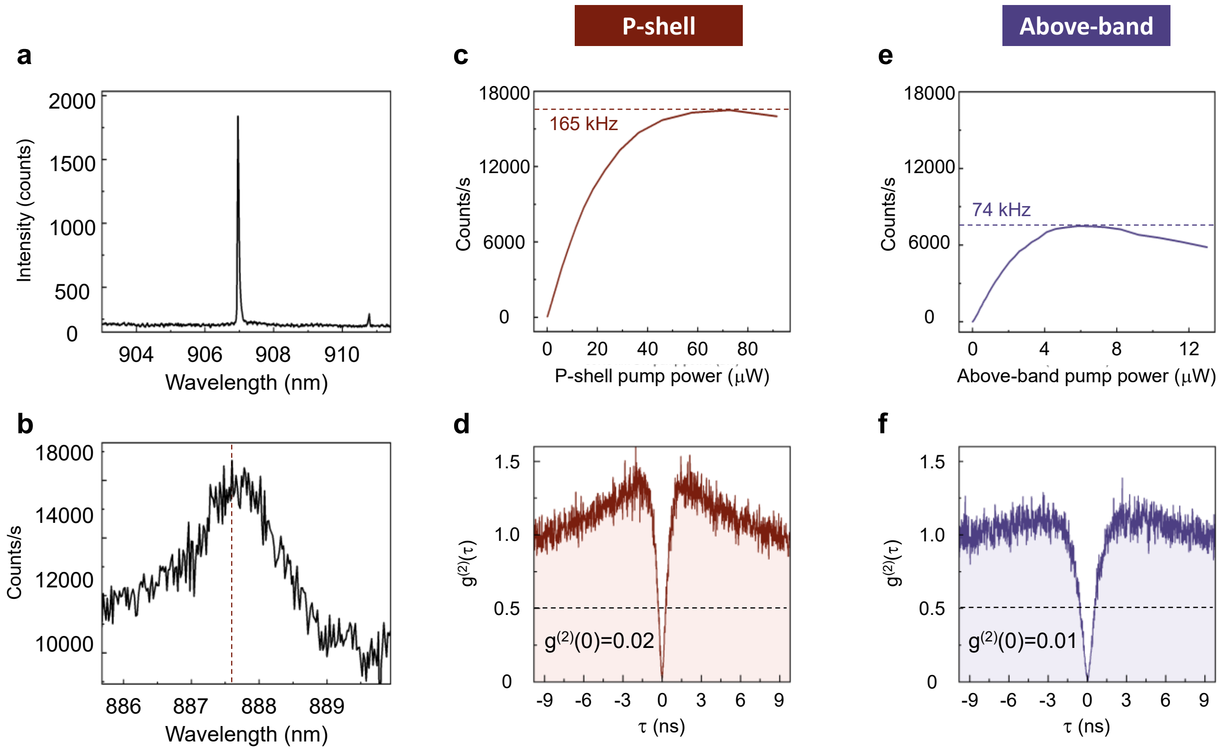

Figure S5 P-shell excitation of a single-photon source based on a topological bulk cavity. a PL spectrum of a QD emission in a bulk cavity. **b** PLE spectrum of the QD emission in (**a**). **c-d** Power-dependent count-rate (**c**) and auto-correlation measurement (**d**) under *p*-shell excitation at 887.4 nm. **e-f** Power-dependent count-rate (**e**) and auto-correlation measurement (**f**) under above-band excitation at 780 nm.

S7. Polarization-resolved measurements on QD1

To conclusively identify the excitonic transitions in QD1 (PL spectrum in Fig. 3b), we conducted polarization-resolved spectroscopy, as shown in Fig. S6a-b. By implementing a $\lambda/2$ plate and linear polarizer in the detection path, we observed that while the neutral exciton (X) and biexciton (XX) emissions exhibited characteristic anticorrelated spectral shifts with polarizer rotation, the charged exciton (X*) emission remained spectrally stable, confirming its charged nature. Further quantum optical characterization reveals an X* radiative lifetime of 786 ps (Fig. S6c) and excellent single-photon purity ($g^{(2)}(0) = 0.04$) (Fig. S6d).

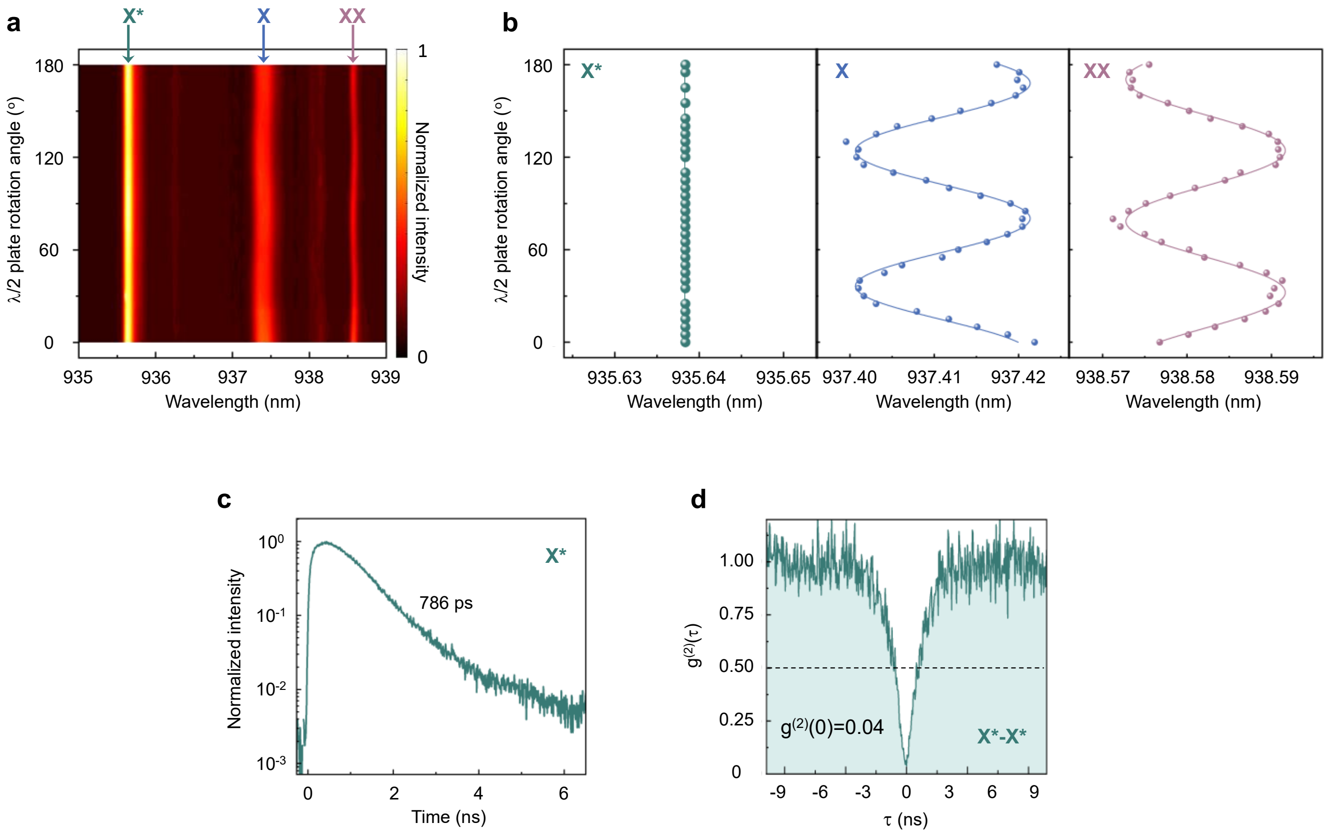

Figure S6 Polarization-resolved measurements on QD1. a Polarization-resolved PL map for the X^*, X and XX emission lines. **b** Polarization-resolved analysis of the X^*, X and XX. **c** Lifetime measurement (**c**) and auto-correlation measurement (**d**) for X* transition.

S8. Topological bulk states under varied cavity sizes

As discussed in the manuscript, bulk states in the topological cavities are confined by band-inversion-induced reflection and the in-plane mode extension is restricted by the topological interface. Thus, size of the bulk state is only determined by period number of the intracavity PC. As shown in Fig.S7a, the mode size of the dipole-like bulk state 1 increases as the intra-cavity period number increases. The increased real-space size facilitates robust QD–cavity coupling which is insensitive to QD position.

Moreover, enlargement of the topological bulk cavity-dimension enhances the quality factor to mode volume ratio, $Q\ V_m^{-1}$, leading to significant improvement of the Purcell factor. In topological bulk cavity, light waves around the center of Brillouin zone (Γ point) get reflected at the topological interface. Expanding the cavity size tightens the momentum-space distribution of the bulk mode to the Γ point due to the uncertainty principle, resulting in a stronger cavity feedback. Consequently, while the mode volume increases with larger PC period number, the Q-factor also rises due to the enhanced in-plane confinement. Crucially, the ratio of $Q\ V_m^{-1}$, which governs the Purcell enhancement, increases with larger PC period. As confirmed by simulation results in the Fig. S7b, $Q\ V_m^{-1}$ increases monotonically with increased cavity size. By doubling the PC period number per cavity edge (from 6 to 12), we observe a 2.6-fold increase in the Purcell factor.

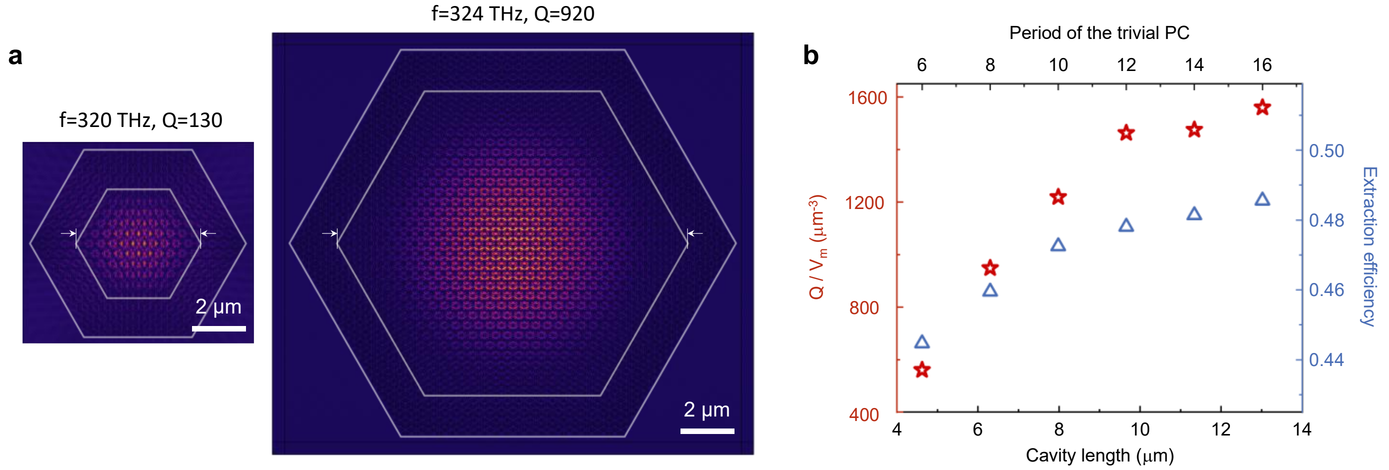

Figure S7. Dipole-like bulk state 1 in topological cavities with varied cavity sizes. a Simulated $|\mathbf{E}|^2$ distribution of bulk mode 1 in two topological cavities with different cavity sizes. The cavity lengths (indicated by white arrows) are 4.62 μm (left) and 13.02 μm (right) respectively, while layers of the cladding areas remain the same. The white hexagons represent the cavity contours. **b** Simulated $Q\ V_m^{-1}$ and extraction efficiency of the bulk cavity versus period of the trivial PC or the cavity length. These simulation results are obtained from ***suspended*** photonic structures without the Au/SiO_2 reflector, which fundamentally limits the maximum extraction efficiency under 50%.

S9. Spatial distribution of the Purcell factor

The topological bulk mode exhibits a position-dependent local distribution with an extended field envelope, enabling a statistically significant probability of observing pronounced Purcell enhancement. In the following, we will introduce the spatial distribution of the topological bulk mode in detail.

Firstly, the spatial dependence of simulated Purcell factor in a unit cell of the trivial PC inside the bulk cavity is shown in Fig. S8a-b. The region with Purcell factor above $0.8\times F_{\mathrm{p}}^{max}$ ($0.45\times F_{\mathrm{p}}^{max}$) covers ~15% (~47%) of the GaAs area, indicated by the dark (light) blue contour. In other words, despite of the local position-dependence, a promising Purcell enhancement can be expected with high likelihood for the case of a unit cell.

Secondly, the overall profile envelope of the cavity mode is smooth and gently varying. As discussed in Fig. S7, the in-plane extension of the topological bulk mode is restricted by the topological interface, thus the size of the bulk mode is only determined by period number of the intracavity PC. Consequently, tolerance of QD positioning in the whole bulk cavity can be achieved by increasing the number of periods of intracavity lattice. For example, by increasing the PC period number per cavity edge to 16, our simulation shows an extended mode distribution with a FWHM of ~5.6 μm shown in Fig. S8c. In this enlarged topological bulk cavity, the region with promising Purcell factor above 8 (4) can cover ~21% (~41%) of the 82 μm^2 GaAs area of the bulk cavity, as shown in Fig. S8d.

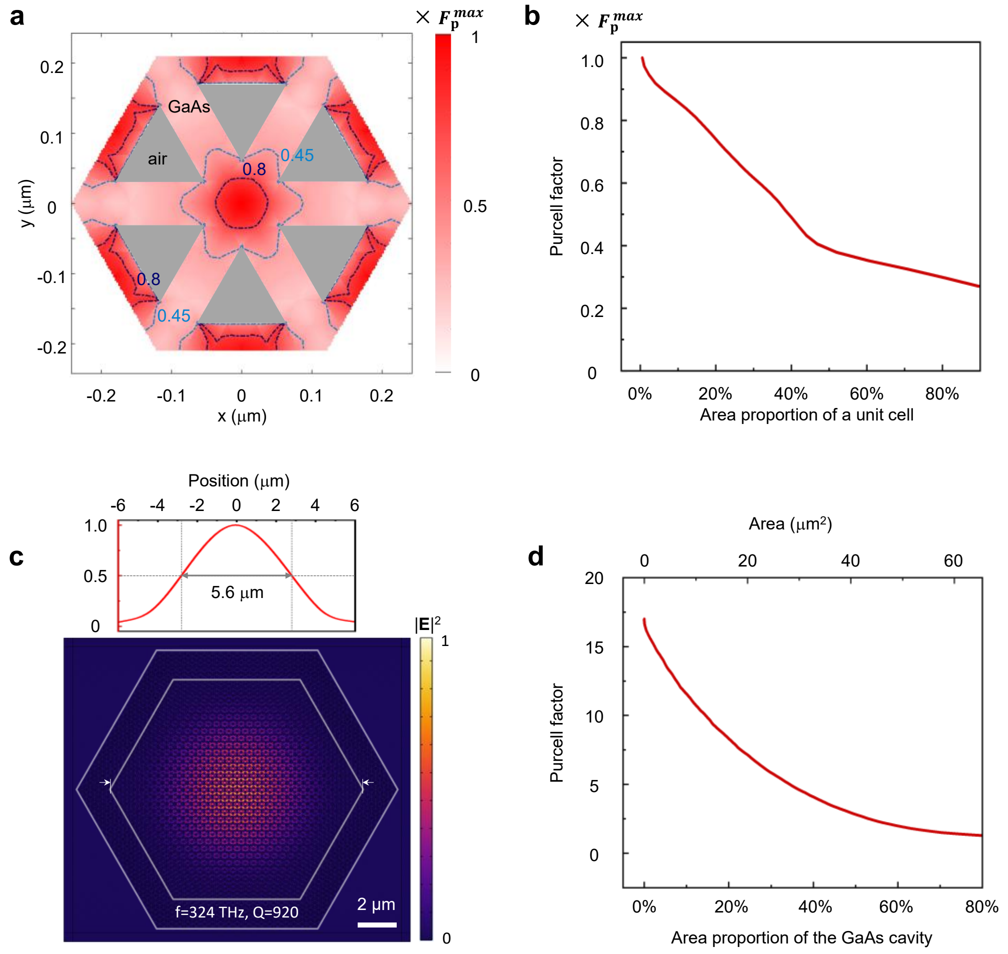

Figure S8. Spatial distribution of the Purcell factor. a Simulated Purcell factor in a unit cell of the PC in the topological bulk cavity. The dashed contour indicates areas with Purcell Factor $>0.8\times F_p^{max}$ or $0.45\times F_p^{max}$, where F_p^{max} is the maximum Purcell factor in this unit cell. **b** Fractional area coverage of distinct Purcell factor contours in a GaAs unit cell. **c** Simulated $|\mathbf{E}|^2$ distribution of bulk mode in a topological cavity with cavity lengths (indicated by white arrows) are 13.02 μm. The white hexagons represent the cavity contour. Inset: Envelope of the $|\mathbf{E}|^2$ field with a FWHM of 5.6 μm. **d** Fractional area coverage of distinct Purcell factor contours in the GaAs cavity in (c).

S10. Topological bulk cavity integrated with highly efficient reflector

Fig. S9 illustrates the optimized topological bulk cavity integrated with a highly efficient reflector. In the optimized device in Fig. S9a, the GaAs slab with etched PC patterns is sitting on a reflector composed of 300-nm SiO_2 and 200-nm gold (Au), to effectively suppress the downwards photon leakage and therefore improve the extraction efficiency. This optimized device can be fabricated by first depositing SiO_2 and Au onto the QD wafer, then removing GaAs substrate and AlGaAs sacrificial layer, and finally etching the PC structure. The structure parameters of the optimized topological bulk cavity and the field distribution of bulk state 1 are shown in Fig. S9b. Development of this optimized device is currently in progress.

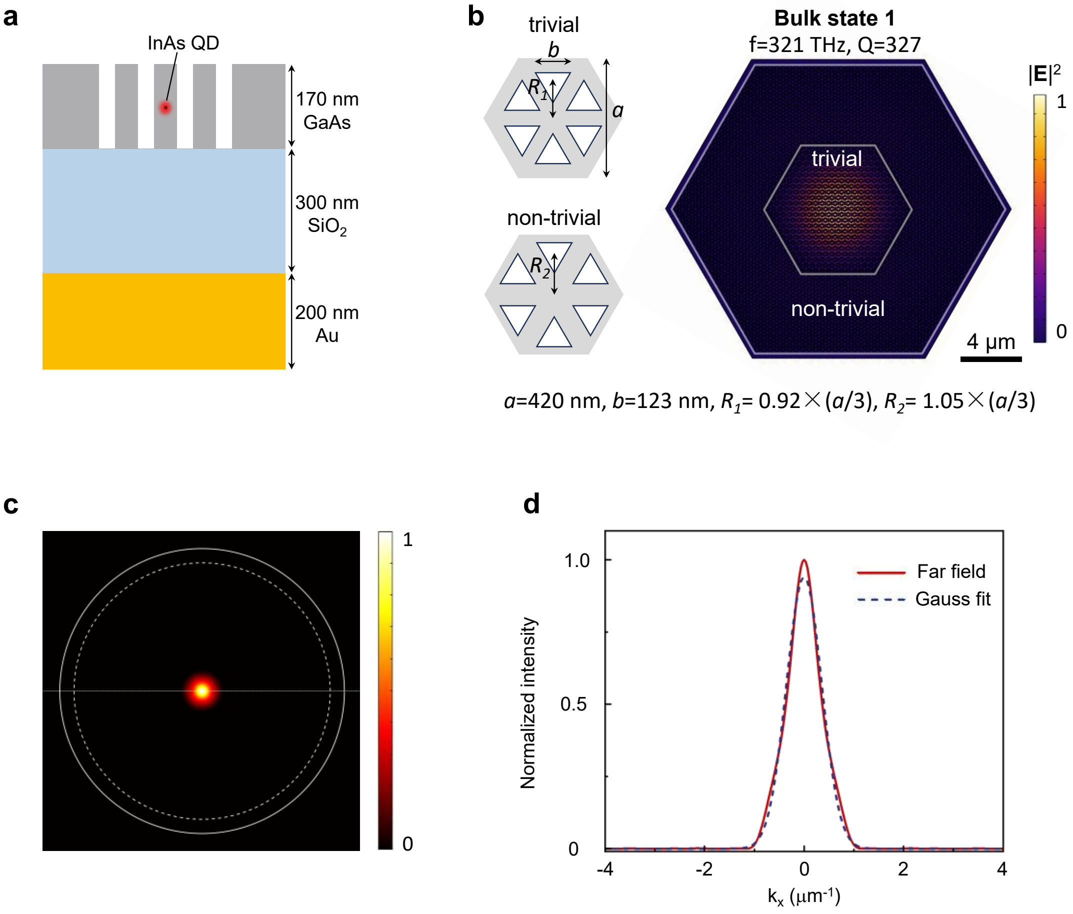

Figure S9 Optimized cavity structure on highly efficient reflector discussed in Fig. 5. a Cross-sectional schematic of the optimized structure. **b** Left: Unit cells of trivial (intracavity) and non-trivial (cladding) PCs. Right: Simulated $|\mathbf{E}|^2$ distribution of dipole-like bulk state 1 in the optimized structure. **c** Simulated angle-resolved far-field distribution of bulk state 1 in the optimized structure. Solid and dashed circles indicate the light cone and NA (=0.9) of the collection objective, respectively. **d** Far-field profile along the cut-line in Fig. S6c and the corresponding Gauss fit.
